# Transportation of Critically Ill Patients on Extracorporeal Membrane Oxygenation

**DOI:** 10.3389/fped.2016.00063

**Published:** 2016-06-13

**Authors:** L. Mikael Broman, Björn Frenckner

**Affiliations:** ^1^ECMO Center, Karolinska University Hospital, Stockholm, Sweden

**Keywords:** extracorporeal membrane oxygenation, interhospital transportation, critically ill patients, intensive care

## Abstract

Extracorporeal membrane oxygenation (ECMO) may be a life-saving procedure for patients with severe reversible pulmonary or cardiac failure or for patients in need for a bridge to transplantation. ECMO is provided by specialized centers, but patients in need of ECMO are frequently taken care of at other centers. Conventional transports to an ECMO center can be hazardous and deaths have been described. For this reason, many ECMO centers have developed transport programs with mobile ECMO. After request, the mobile team including all necessary equipment to initiate ECMO is sent to the referring hospital, where the patient is cannulated and ECMO commenced. The patient is then transported on ECMO to the ECMO facility by road, helicopter, or fixed-wing aircraft depending on distance, weather conditions, etc. Eight publications have reported series of more than 50 transports on ECMO of which the largest included over 700. Together, these papers report on more than 1400 patient transports on ECMO. Two deaths during transport have occurred. A number of other adverse events are described, but without effect on patient outcome. Survival of patients transported on ECMO is equivalent to that of non-transported ECMO patients. It is concluded that long-, short-distance interhospital transports on ECMO can be performed safely. The staff should be experienced and highly competent in intensive care, ECMO cannulation, ECMO treatment, intensive care transport, and air transport medicine.

## Background

Extracorporeal membrane oxygenation (ECMO) may be a life-saving procedure for patients with refractory, severe respiratory, and/or circulatory failure. Only specialized centers provide ECMO, and patients, therefore, may have to be transported for treatment. Patients eligible for ECMO are, in general, in an unstable condition supported by maximal ventilator support and other intensive care salvage therapies. A conventional transport may be hazardous, and deaths have been described (1, 2). Cannulating the patient at the referring hospital and transporting the patient on ECMO was first described by Bartlett et al. (3). The concept of traveling to the referring hospital with a portable ECMO system, cannulating the patient at the referring hospital and transporting the patient back to your own institution on ECMO was then further developed by Cornish (4, 5). Since then, the number of centers providing transports on ECMO has increased, but it was not widely performed until the late 2000s (6). The University of Michigan, Ann Arbor, recently reported their experience from the transport of 221 patients on ECMO between 1990 and 2012 (6). This paper also included a review of the literature with 27 published articles describing the ECMO transport of altogether 643 patients. The experience in ECMO transports differs among centers, and today, only 4 centers have reported a total of more than 100 transports, University of Arkansas for Medical Sciences College of Medicine, Little Rock (7), The University of Michigan, Ann Arbor (6), Columbia University Medical Center, New York (8), and Karolinska University Hospital, Stockholm (9), of which the latter center had performed over 700 transports up to 2015.

Extracorporeal Life Support Organization (ELSO, Ann Arbor, MI, USA, www.elso.org) is a worldwide consortium of units providing ECMO with over 400 member centers (personal communication Peter Rycus, ELSO Registry, Ann Arbor, MI, USA). Besides from maintaining a registry of ECMO treatments, another mission of ELSO is to provide different guidelines associated with ECMO treatment. Recently, guidelines for ECMO transport were published (10).

## Methods

### Logistics

The mission is to transport an extremely sick and unstable patient on ECMO from the referring hospital to an ECMO center, which, in most instances, is the transport teams own facility. In most cases, when the patient is not already on ECMO, the commitment involves a final assessment of the patient, cannulation, and initiation of ECMO at the referring hospital in addition to the transport. This is referred to as a primary transport. In some cases, the patient might already be on ECMO at the referring hospital. This may be the case if the referring hospital has the capability of cannulation and initiation of ECMO or if the patient is transferred between different ECMO centers, often thoracic intensive care units. These transports are referred to as secondary transports (10). It is obvious that a transport team having the capability for primary transports also has the capability of secondary transports. Therefore, this paper will mainly focus on primary transports.

An ECMO transport consists of three parts, namely, (1) transport to the referring hospital with personnel and equipment, (2) procedures at the referring hospital (final assessment of the patient, cannulation, stabilization, etc.), and (3) transport back to your own facility or to another ECMO center. In most instances, it starts with a phone call from the referring hospital, and a decision is made to launch the mobile ECMO team. To minimize the time to reach and secure, the patient is the major priority in planning the primary transport. On the other hand, when transporting the patient on ECMO to the ECMO facility, patient safety is the overriding priority, and time is of secondary importance (10).

Once the decision is made to launch the transport team for a primary transport, the team members must be summoned, necessary equipment packed, and transport vehicle/vehicles be organized.

### Transport Vehicle

Ground ambulance, helicopter, and fixed-wing aircraft are the three options that have been used for ECMO transports. Distance of transport, weather conditions, and availability will decide which vehicle is used. Transport on fixed-wing aircraft for obvious reasons also involves ground transport to and from the airport at both ends with loading and unloading. In hospitals equipped with helicopter pads, the patient is loaded directly into the helicopter and ground transport is unnecessary in good weather conditions. Some helicopters have equipment for operating in instrument flight rules (IFR) conditions, which enables flight in worse weather, but will then have to depart from and arrive to local airports. In the ELSO guidelines, ground transport is recommended for distances up to 400 km (250–300 miles) and helicopter transport for distances up to 650 km (300–400 miles). Fixed-wing aircraft can transport any distance (Table 1) (10).

**Table 1:** **Properties of ground ambulance, helicopter, and fixed-wing aircraft [from ELSO guidelines (10)]**.

	Ground ambulance	Helicopter	Fixed-wing aircraft
Space for team and equipment	Sufficient (4–5 team members)	More limited (3–5 team members)	Variable (≥4 team members)
Noise	Relatively little	Very loud	Loud
Distance for reasonable transport times	Up to 400 km (250–300 miles)	Up to 650 km (300–400 miles)	Any distance
Weight limitations	Unlimited	Limited (impacted by distance and weather)	Variable (depending on aircraft and conditions)
Loading and securing equipment and ECMO circuit/patient	Relatively easy	Relatively easy	Variable (depending on equipment and aircraft model)
Cost	++	+++	++++

Transport vehicles/aircraft should have electrical supply sufficient for the ECMO pump, heater, and all other electrical equipment used in the transport. Oxygen supply must also be provided for the entire transport. Climate control, suction, and adequate lightning are also essential.

Normally, the same transport vehicle is used both for transport to the referring hospital and for transport back to the ECMO facility (10). However, occasionally, it is quicker to reach the referring hospital with another vehicle, i.e., if the transport vehicle is not available or if the patient is to be transported to the ECMO facility with a mobile intensive care unit (MICU) in which cases an emergency vehicle may be used (9). Other examples of vehicles transporting the team to the referring hospital are taxi-cab, taxiplane, helicopter, and regular aircraft MD-80 (11).

### Equipment

The medical equipment used in ECMO transports is in principle the same as in in-house ECMO treatment, although unique aspects and limitations in the transport environment may impose specific requirements. For all air transports, the individual components and the assembled equipment must meet specific air worthiness criteria and be approved by national regulatory agencies. The emission of electromagnetic interference must be low enough not to interfere with flight controls, and adjustments of the equipment may be necessary. Furthermore, it must sustain vibrations and acceleration/deceleration forces during takeoff, the flight, and landing.

Transports have been performed both with roller and centrifugal pumps. Roller pumps were more common in the earlier era of transports (11–14) and later on centrifugal pumps were preferred by most centers (6, 8, 9, 15–18). Today, the latter is recommended by ELSO due to improved functionality and safety in combination with a shorter circuit (10). The membrane oxygenator and tubing size should be appropriate for the patient size.

The transport stretcher or sled varies between centers. Many centers have developed their own custom made system, where the ECMO circuit components and stretcher are assembled in one unit (7, 12, 14) making the tubing shorter and minimizing the risk for tubing kinking, etc., during loading and unloading the patient into and out of the transport vehicle. A commercially available ECMO transport system is in fact visible on the ELSO web site (www.elso.org). Other centers have used separate stretcher and transport ECMO carts, which require longer tubing but minimize the weight of the stretcher with the patient (9). All items must be securely fastened in the transport vehicle. When working in a pre-hospital milieu with these complex transfers and differently designed vehicles, the experience of the mobile ECMO team comes in play for safe unloading/loading. In many cases, the supportive ambulance/air craft staff is out of their normal context with limited knowledge in these kinds of patients and technology.

Once the transport team has left the ECMO center, they must be self-sufficient with respect to all ECMO specific supplies. For example, disposables are needed to assemble the ECMO circuit, and a spare emergency ECMO circuit has to be brought in case of unexpected adverse events. Connectors, extra pump head and oxygenator, and spare tubing may be required. Surgical equipment for cannulation should be brought by the transport team if it is not assured that it is available at the referring hospital. This includes sterile surgical instruments, surgical disposables like sutures, etc., surgical dressing, and head lamp. Cannulas are packed in suitable sizes according to the weight of the patient.

Electrical cautery device and bedside ultrasound device for assistance with cannulation is generally available at the referring hospital. Blood products necessary for initiation of ECMO and for transport back up has to be immune-compatible and hence provided by the referring facility. Devices for assessment of blood gases and anticoagulation monitoring, i.e., ACT machine, must be brought. In addition to this, all equipment and supplies necessary during conventional transports of intensive care patients are needed: transport ventilator, patient monitoring device, infusion pumps, and pharmaceuticals.

Using check lists for necessary equipment are recommended prior to departure from the ECMO center to the referring hospital (10). Much of the equipment can also be stored in prepacked sealed and signed bags, in order to enhance departure (9). There should be no acceptance tampering on the patient (or staff) safety measures. The transport must be as safe for the patient as ever possible by the surrounding organization.

### Personnel

Different centers have very different composition of their transport teams, which mainly depends on different competences, duties, and traditions for the respective professions. For example, in most centers, the ECMO machine is primed by a perfusionist, but in other centers this may be accomplished by a physician or a nurse (R.N.) with special education. In many centers, the cannulation is always performed by a surgeon, but in other, the intensivist may do percutaneous cannulations (19, 20). The ventilator may be managed by a respiratory therapist (R.R.T.), a doctor or a nurse, and so on. When configuring a transport team, it is important to list all responsibilities that are needed for the mission. These will include capability to evaluate the patient and ECMO indication, to cannulate the patient, to prime the ECMO circuit, to initiate ECMO treatment, to manage the critical ill patient now on ECMO, including the ECMO circuit, ventilator, medications, and anticoagulation, and to handle common or any unforeseen problems and complications. All responsibilities must be met by experienced personnel, as there will be no back up at hand.

An example of how a transport team can be composed is given in the ELSO guidelines (10). This team consists of a cannulating physician, a surgical assistant, an ECMO physician, an ECMO specialist, and a transport R.N./R.R.T. Other examples are the University of Michigan transport team consisting of a critical care surgeon, a critical care fellow, and two ECMO specialists (6), the Arkansas Children’s Hospital transport team with a pediatric cardiac surgeon, a surgical assistant, an intensive care physician, and an ECMO coordinator (7), Columbia University Medical Center transport team with one cardiothoracic surgeon, one surgical fellow, two perfusionists, and two critical care paramedics (8), and the Karolinska University Hospital transport team with one surgeon, one ECMO intensivist, one ECMO specialist, and occasionally one scrub nurse (9). The Wilford Hall Medical Center has used larger transport teams consisting of 10–15 persons (21).

### Special Situations

#### International Transports

It is every staff member’s responsibility to carry a valid passport and, during the early contacts with the referring hospital, ask them to do what is manageable in retrieving the patients’ passport. It is also advised to bring internationally viable currency for emergency situations. You might get stranded, with or without the patient.

In international transfers, the transport team must be aware of the standard of electrical sockets and gas outlets in the current country. Compliable gas and electrical adaptors must be brought.

When planning for extremely long transports (intercontinental transports), extra staff should be considered. Functions in the ECMO team that will be active for a majority of the time should be up-numbered. Functions that will be active only for a shorter part may not have to be increased in number. For example, in our organization, the cannulating surgeon is active during final assessment of the patient and during the cannulation procedure. When ECMO commenced that function will step back and be “stand-by” for surgical emergencies. During intercontinental secondary transports, it should be advised to bring a surgical competence for patient safety reasons, even though the workload is estimated to nil. It cannot be emphasized enough that it is important to assure that enough supplies, medication, oxygen, etc., are available for the whole transport. The amount of oxygen brought should cover for a significant time delay and increase in patient oxygen demand.

#### Environmental/Climate Issues

When patients are transported in an arctic environment, it is unadvisable and may be hazardous to load the patient into the aircraft outdoors in extreme cold. In such instances, the transport team may use a heated hangar to load (or unload) the patient into the aircraft. In an environment of −20 to −30°C (−4 to −22°F), the smaller patient will be hypothermic within a few minutes, and infusion lines and stopcocks freeze within 30 s (unpublished data). In our experience, plastic materials get fragile and break very easily, even larger devices, such as ventilator tubing. When working in cold environment and no hangar is available, it is essential to plan every move beforehand with all the support at hand. If any trouble, it is better to return to the warm ambulance and re-plan, than to get stuck in the door to the aircraft.

#### Issues Concerning Working Environment

The working conditions during ambulance, helicopter, or fixed-wing transports are submitted to noise, vibrations, and limitations in both working space and light. Alarms might not be heard, and the sector from which the monitoring can be easily observed is limited, putting the clinical experience of the ECMO team members in focus. In damp light conditions, the color of the venous and arterial ECMO tubing will be hard to discriminate. Working space may be further limited due emergency equipment has be reachable in case of emergency or adverse event. Laboratory devices may be sensitive to cold and fail.

It would be advised to bring some drinking water and a small box of high energy containing solid food stuffs, in case of delay or personnel calorie fatigue. An average primary ECMO transport may in our experience require 7 h out of hospital but may for several reasons be stretched to twice that time or more.

## Results

There are numerous publications on transportation of critically ill patients on ECMO including small series and case reports. Eight unique publications report materials exceeding 50 patients (21–24) of which four report 100 or more patients (6–9) (Table 2). The patients in the publication by Foley et al. in 2002 (12) are included in the same group’s later publication (6) and are, therefore, not listed separately in Table 2. These centers have together published their experience from transportation of over 1400 patients. Two deaths have occurred. Both these patients were on VV ECMO and suffered unexpected cardiac failure (6, 9). Survival to discharge was the same in patients transported on ECMO as in ECMO patients, in large.

**Table 2:** **Transport data from eight papers reporting over 50 transports**.

Reference	No. of patients	Adverse effects	Age groups	Vehicle	Distance (km)
Coppola et al. (21)	68	No death	Neo/Ped	Ground/Fixed-Wing	13–12,070
		Several adverse effects			
Clement et al. (7)	112	No death	Neo/Ped	Ground/Heli/Fixed-Wing	NR
Beurtheret et al. (22)	75	No death	Adult	Ground	4–243
		One pump malfunction			
Roch et al. (24)	85	No serious complications	Adult	NR	NR
Bryner et al. (6)	221	1/221 death	Neo/Ped/Adult	Ground/Heli/Fixed-Wing	Mean 172
					Max 3,100
Schopka et al. (23)	68	No death	Adult	Ground/Heli	5–300
Biscotti et al. (8)	100	No adverse effects	Adult	Ground/Fixed-Wing	4–10,700
Broman et al. (9)	700 (322)^a^	1/700 death	Neo/Ped/Adult	Ground/Heli/Fixed-wing	7–13,447
		Several adverse effects			

*Neo, neonatal patients; Ped, pediatric patients; Adult, adult patients; NR, not reported; Ground, ground ambulance; Heli, helicopter; Fixed-wing, fixed-wing aircraft*.

*^a^The material includes over 700 transports and a detailed report is given on 322 transports during a 4-year period*.

### Adverse Events

There are no publications regarding adverse events or complications during ECMO transports. Experience from 395 transfers on ECMO over five consecutive years was presented as a poster at EURO-ELSO 2015 (25). Ninety-four percent of these could be scrutinized and adverse events of any kind occurred in 30.8% of the transports. In 6.2%, more than one complication was reported. Most common were event related to the patient (28.2%), of which loss of tidal volume accounted for 11.5%. Second most common were circulatory problems, including bleeding (5.6%). Equipment-related problems occurred in 19 cases of which heater failure in 4. Three patients experienced hypothermia, intravenous lines froze on two transports, and two traffic accidents passed during the study period. No deaths occurred even though one oxygenator clotted with total cessation of ECMO blood flow in an ambulance. That oxygenator showed no prewarning, was exchanged quickly, and the patient suffered no sequelae.

In attempt to categorize the severity of adverse events during interhospital ECMO transports, we recently conducted a retrospective investigation of 452 transports between 2010 and 2015 (26). Approximately 60% of these transports engaged both aircraft and ground ambulance. The remainder utilized only a ground ambulance resource. One hundred sixty-five adverse events were taken to the charts in 115 (25.4%) of these transports. In 6% of the transfers, more than one incident occurred. Fifty-seven percent of these events exposed the patient and/or ECMO team to a *not negligible risk* as categorized in Table 3. It should be emphasized that risk situations do occur during transfers on ECMO, since these sometimes engage more than 40 different people outside of the ECMO transportation team. These “bystanders,” referring hospital, air-, and ambulance coordinators, services, and staff, are often only vaguely aware of the demands from such complex transports. In 4% of the transports, the lack of knowledge and adaptation by the ambulance service exposed both patient and transport team to risk. In 2%, an *Immediate-threat-of-life* situation was observed. These events though, related directly to the ECMO circuit and patient status, no deaths occurred in this survey.

**Table 3:** **Complications during 452 ECMO transports from a single center between 2010 and 2015 (26)**.

Immediate threat	*n*	High risk	*n*	Variable risk	*n*
Clotting of ECMO circuit	2	Loss of tidal volume	44	Airport logistics/delay	4
Inadequate ECMO (VV to VA)	2	Bleedings	12	Wrong ambulance/delay	3
System/pump change	2	Circulatory instability	7	Ambulance utility malfunction	3
Oxygenator clot	2	Broken ventilator circuit	2	Ambulance traffic accident	2
Cannula clot	1	Reload in ambient temp	2	ECMO system forgotten	1
IV line/air into circuit	1	Broken sweep gas supply	1	ECMO pump head forgotten	1
		Power supply roller pump	1		
		Recirculation (VV ECMO)	1		
Sum	10		70		14
Fraction of total (*n* = 452)	2.2%		15%		3.1%

## Discussion

To improve outcome and optimize health-care resources, it has been proposed to consolidate ECMO treatment to high-volume centers (27). Such systems require an effective ECMO retrieval organization.

There are no published data to support the minimum transport numbers a mobile ECMO team should perform. Concerning adults, a consensus statement from the International ECMO Network proposed 20 cases on an annual basis (19, 27). For neonatal and pediatric patients, no data are at hand, though a similar figure has been recommended concerning annual ECMO runs to increase survival rate compared with low volume intensive care units (20, 28). Of today, 20 annual cases seem to be the lower number for an adequate learning curve and to maintain ECMO competence.

Data based on the ELSO Registry (21, 29) showed that ECMO centers with more than 30 annual adult ECMO had a significantly lower ECMO mortality than units with fewer than 6 cases per year. This volume–mortality association might favor a policy to continue and expand treatment at the experienced centers or even to centralize ECMO treatment, instead of starting an ECMO program at another hospital (19, 27). Others have also expressed support of transfers to regional ECMO centers (16, 18, 22, 30–32) as a few nations have arranged their ECMO resources in such manner (The United Kingdom, Australia, and Italy).

Only a few studies have reported morbidity figures. The population exposed to an ECMO transport has about the same mortality rate as the corresponding non-transferred patients at a given ECMO center (7, 11, 12, 18, 30). The future rationale would be that one well-trained transport organization performs ECMO transport at a high-volume ECMO unit, as part of their service for a particular region (18, 19, 22, 27, 30, 32). This does not disqualify, but rather encourage, the lower-volume hospitals to commence ECMO in an emergency patient as agreed with the regional large-volume unit that subsequently retrieves the patient for continuance of ECMO treatment at the regional ECMO center (19, 20, 27). Some Finnish university hospitals less experienced in ECMO proceed in this manner. In a 4-year data survey from our unit, 13 patients died before ECMO was commenced (unpublished data). A few of these lives might have been saved in a system where an ECMO resource had been faster bedside for cannualtion and initiation of ECMO.

## Conclusion

Transports on ECMO are generally safe, if conducted by experienced staff. High-risk or *Immediate-threat-of-life* situations will occur. The later needs to be taken care of within seconds, demanding highly trained personnel and the transport to be organized accordingly.

## Author Contributions

LB and BF have together analyzed the literature, written, and revised the manuscript.

## Conflict of Interest Statement

The authors declare that the research was conducted in the absence of any commercial or financial relationships that could be construed as a potential conflict of interest.

## References

[1] Peek GJ Mugford M Tiruvoipati R Wilson A Allen E Thalanany MM Efficacy and economic assessment of conventional ventilatory support versus extracorporeal membrane oxygenation for severe adult respiratory failure (CESAR): a multicentre randomised controlled trial. Lancet (2009) 374(9698):1351. 10.1016/S0140-6736(09)61069-2 19762075

[2] Boedy RF Howell CG Kanto WP Jr. Hidden mortality rate associated with extracorporeal membrane oxygenation. J Pediatr (1990) 117(3):462. 10.1016/S0022-3476(05)81098-4 2391605

[3] Bartlett RH Gazzaniga AB Fong SW Jefferies MR Roohk HV Haiduc N. Extracorporeal membrane oxygenator support for cardiopulmonary failure. Experience in 28 cases. J Thorac Cardiovasc Surg (1977) 73(3):375. 839827

[4] Cornish JD Gerstmann DR Begnaud MJ Null DM Ackerman NB Inflight use of extracorporeal membrane oxygenation for severe neonatal respiratory failure. Perfusion (1986) 1(4):281 10.1177/026765918600100408

[5] Cornish JD Carter JM Gerstmann DR Null DM Jr. Extracorporeal membrane oxygenation as a means of stabilizing and transporting high risk neonates. ASAIO Trans (1991) 37(4):564. 1768489

[6] Bryner B Cooley E Copenhaver W Brierley K Teman N Landis D Two decades’ experience with interfacility transport on extracorporeal membrane oxygenation. Ann Thorac Surg (2014) 98(4):1363. 10.1016/j.athoracsur.2014.06.025 25149055

[7] Clement KC Fiser RT Fiser WP Chipman CW Taylor BJ Heulitt MJ Single-institution experience with interhospital extracorporeal membrane oxygenation transport: a descriptive study. Pediatr Crit Care Med (2010) 11(4):509. 10.1097/PCC.0b013e3181c515ca 20595821

[8] Biscotti M Agerstrand C Abrams D Ginsburg M Sonett J Mongero L One hundred transports on extracorporeal support to an extracorporeal membrane oxygenation center. Ann Thorac Surg (2015) 100(1):34. 10.1016/j.athoracsur.2015.02.037 25912741

[9] Broman LM Holzgraefe B Palmér K Frenckner B The Stockholm experience: inter-hospital transports on extracorporeal membrane oxygenation. Crit Care (2015) 19:278 10.1186/s13054-015-0994-6 26160033 PMC4498561

[10] ELSO. Guidelines for ECMO Transport Ann Arbor: ELSO. (2015). Available from: http://www.elso.org/Resources/Guidelines.aspx

[11] Linden V Palmer K Reinhard J Westman R Ehren H Granholm T Inter-hospital transportation of patients with severe acute respiratory failure on extracorporeal membrane oxygenation – national and international experience. Intensive Care Med (2001) 27(10):1643 10.1007/s001340101060 11685306

[12] Foley DS Pranikoff T Younger JG Swaniker F Hemmila MR Remenapp RA A review of 100 patients transported on extracorporeal life support. ASAIO J (2002) 48(6):612. 10.1097/00002480-200211000-00007 12455771

[13] Heulitt MJ Taylor BJ Faulkner SC Baker LL Chipman CW Harrell JH Inter-hospital transport of neonatal patients on extracorporeal membrane oxygenation: mobile-ECMO. Pediatrics (1995) 95(4):562. 7700759

[14] Wilson BJ Jr Heiman HS Butler TJ Negaard KA DiGeronimo R. A 16-year neonatal/pediatric extracorporeal membrane oxygenation transport experience. Pediatrics (2002) 109(2):189. 10.1542/peds.109.2.189 11826194

[15] Forrest P Ratchford J Burns B Herkes R Jackson A Plunkett B Retrieval of critically ill adults using extracorporeal membrane oxygenation: an Australian experience. Intensive Care Med (2011) 37(5):824. 10.1007/s00134-011-2158-8 21359610

[16] Haneya A Philipp A Foltan M Mueller T Camboni D Rupprecht L Extracorporeal circulatory systems in the interhospital transfer of critically ill patients: experience of a single institution. Ann Saudi Med (2009) 29(2):110. 10.4103/0256-4947.51792 19318758 PMC2813631

[17] Lucchini A De Felippis C Elli S Gariboldi R Vimercati S Tundo P Mobile ECMO team for inter-hospital transportation of patients with ARDS: a retrospective case series. Heart Lung Vessel (2014) 6(4):262. 25436208 PMC4246845

[18] Raspe C Ruckert F Metz D Hofmann B Neitzel T Stiller M Inter-hospital transfer of ECMO-assisted patients with a portable miniaturized ECMO device: 4 years of experience. Perfusion (2015) 30(1):52. 10.1177/0267659114531611 24743549

[19] Conrad SA Grier LR Scott LK Green R Jordan M. Percutaneous cannulation for extracorporeal membrane oxygenation by intensivists: a retrospective single-institution case series. Crit Care Med (2015) 43(5):1010. 10.1097/CCM.0000000000000883 25746749

[20] Sherren PB Shepherd SJ Glover GW Meadows CI Langrish C Ioannou N Capabilities of a mobile extracorporeal membrane oxygenation service for severe respiratory failure delivered by intensive care specialists. Anaesthesia (2015) 70(6):707. 10.1111/anae.13014 25850687

[21] Coppola CP Tyree M Larry K DiGeronimo R. A 22-year experience in global transport extracorporeal membrane oxygenation. J Pediatr Surg (2008) 43(1):46. 10.1016/j.jpedsurg.2007.09.021 18206454

[22] Beurtheret S Mordant P Paoletti X Marijon E Celermajer DS Leger P Emergency circulatory support in refractory cardiogenic shock patients in remote institutions: a pilot study (the cardiac-RESCUE program). Eur Heart J (2013) 34(2):112. 10.1093/eurheartj/ehs081 22513777

[23] Schopka S Philipp A Hilker M Muller T Zimmermann M Arlt M Clinical course and long-term outcome following venoarterial extracorporeal life support-facilitated interhospital transfer of patients with circulatory failure. Resuscitation (2015) 93:53. 10.1016/j.resuscitation.2015.05.021 26051810

[24] Roch A Hraiech S Masson E Grisoli D Forel JM Boucekine M Outcome of acute respiratory distress syndrome patients treated with extracorporeal membrane oxygenation and brought to a referral center. Intensive Care Med (2014) 40(1):74. 10.1007/s00134-013-3135-1 24170143 PMC7095017

[25] Ericsson A Broman LM Five-Year Follow-Up of Adverse Events During Inter-Hospital Transports on Extracorporeal Membrane Oxygenation. Regensburg, Germany: EuroELSO (2015). 10.1080/10903127.2017.1282561 28166435

[26] Broman LM High-Risk Inter-Hospital Transports on Extracorporeal Membrane Oxygenation. Glasgow, Scotland: EuroELSO (2016).

[27] Combes A Brodie D Bartlett R Brochard L Brower R Conrad S Position paper for the organization of extracorporeal membrane oxygenation programs for acute respiratory failure in adult patients. Am J Respir Crit Care Med (2014) 190(5):488. 10.1164/rccm.201404-0630CP 25062496

[28] Freeman CL Bennett TD Casper TC Larsen GY Hubbard A Wilkes J Pediatric and neonatal extracorporeal membrane oxygenation: does center volume impact mortality? Crit Care Med (2014) 42(3):512. 10.1097/01.ccm.0000435674.83682.96 24164955 PMC4548884

[29] Barbaro RP Odetola FO Kidwell KM Paden ML Bartlett RH Davis MM Association of hospital-level volume of extracorporeal membrane oxygenation cases and mortality. Analysis of the extracorporeal life support organization registry. Am J Respir Crit Care Med (2015) 191(8):894. 10.1164/rccm.201409-1634OC 25695688 PMC4435456

[30] Javidfar J Brodie D Takayama H Mongero L Zwischenberger J Sonett J Safe transport of critically ill adult patients on extracorporeal membrane oxygenation support to a regional extracorporeal membrane oxygenation center. ASAIO J (2011) 57(5):421. 10.1097/MAT.0b013e3182238b55 21869618

[31] Starck CT Hasenclever P Falk V Wilhelm MJ. Interhospital transfer of seriously sick ARDS patients using veno-venous extracorporeal membrane oxygenation (ECMO): concept of an ECMO transport team. Int J Crit Illn Inj Sci (2013) 3(1):46. 10.4103/2229-5151.109420 23724385 PMC3665119

[32] Wiegersma JS Droogh JM Zijlstra JG Fokkema J Ligtenberg JJ. Quality of interhospital transport of the critically ill: impact of a Mobile Intensive Care Unit with a specialized retrieval team. Crit Care (2011) 15(1):R75. 10.1186/cc10064 21356054 PMC3222008

